# Beneficial effects of sulfonamide-based gallates on osteoblasts *in vitro*

**DOI:** 10.3892/mmr.2021.12152

**Published:** 2021-05-13

**Authors:** Li Huang, Pan Jin, Xiao Lin, Cuiwu Lin, Li Zheng, Jinmin Zhao

Mol Med Rep 15: 1149-1156, 2017; DOI: 10.3892/mmr.2017.6142

Following the publication of the above article, an interested reader drew to the authors' attention that three figures in their paper (namely Figs. 2, 4 and 5) appeared to feature panels containing overlapping data. The authors re-examined their original data, and realized that they had made inadvertent errors in the compilation of the data in these figures; specifically, the data shown in the panels for Fig. 2G, Fig. 4C, D and I, and Fig. 5E and I had been selected incorrectly.

The corrected versions of Figs. 2, 4 and 5 are shown below and on the next page. All the authors approve of this corrigendum, and are grateful of the Editor of *Molecular Medicine Reports* for granting them the opportunity to publish this corrigendum. Furthermore, they regret that these errors were introduced into the paper, even though they did not substantially alter any of the major conclusions reported in the paper, and apologize to the readership for any inconvenience caused.

## Figures and Tables

**Figure 2.**
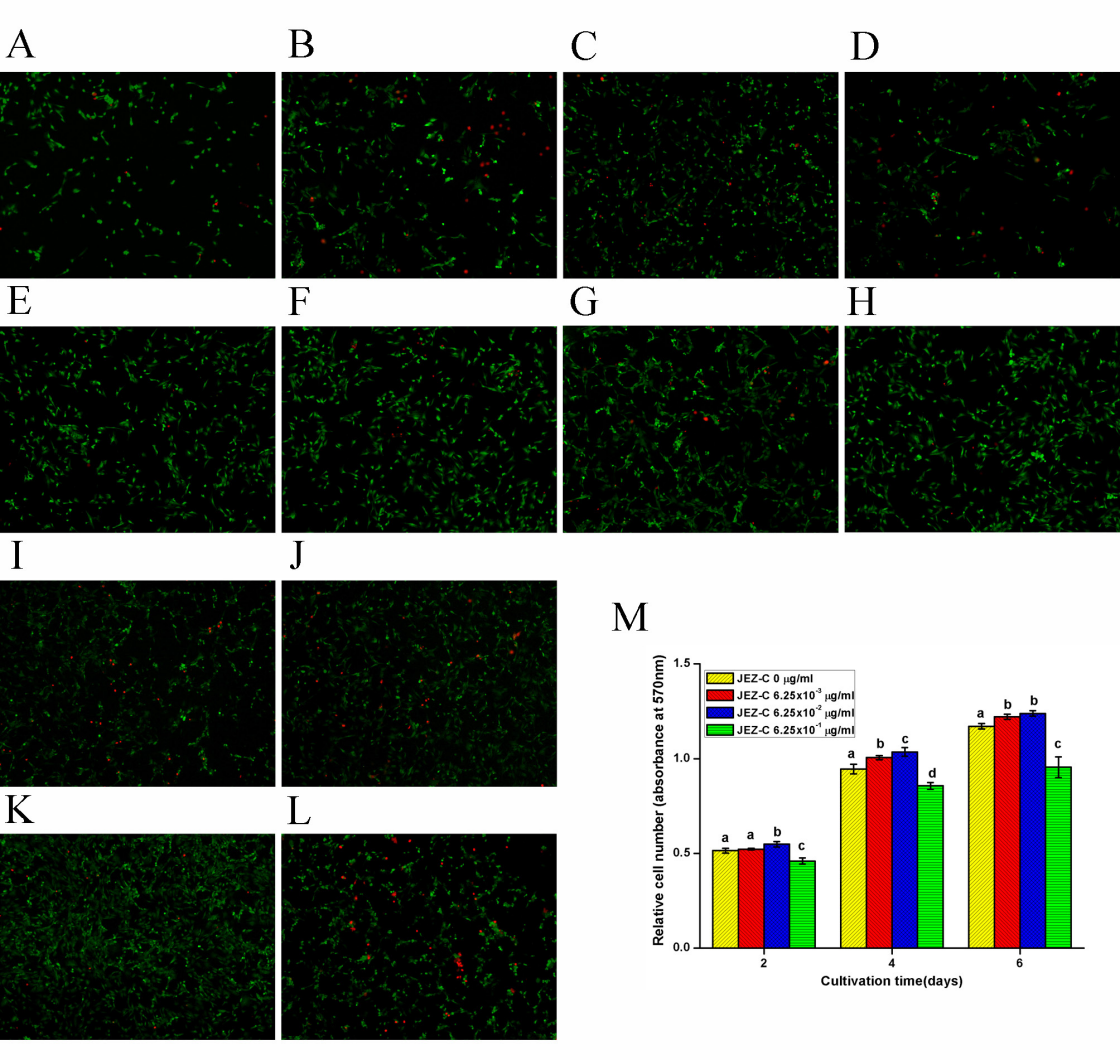
Fluorescein diacetate/propidium iodide staining of osteoblasts cultured with various concentrations of JEZ-C over time. Viable cells were stained green and dead cells were stained red. Staining of osteoblasts treated with (A) 0 µg/ml, (B) 6.25×10^−3^ µg/ml, (C) 6.25×10^−2^ µg/ml and (D) 6.25×10^−1^ µg/ml JEZ-C at day 2. Staining of osteoblasts treated with (E) 0 µg/ml, (F) 6.25×10^−3^ µg/ml, (G) 6.25×10^−2^ µg/ml and (H) 6.25×10^−1^ µg/ml JEZ-C at day 4. Staining of osteoblasts treated with (I) 0 µg/ml, (J) 6.25×10^−3^ µg/ml, (K) 6.25×10^−2^ µg/ml and (L) 6.25×10^−1^ µg/ml JEZ-C at day 6. Staining was markedly strengthened over time in all groups. Cells treated with JEZ-C exhibited stronger staining compared with the control group, particularly at 6.25×10^−2^ µg/ml. Scale bar=200 µm. (M) Relative cell number of osteoblasts treated with various concentrations (0, 6.25×10^−3^, 6.25×10^−2^ and 6.25×10^−1^ µg/ml) of JEZ-C. Cell proliferation was higher in the 6.25×10^−2^ µg/ml group compared with in the other groups. Data are presented as the mean ± standard deviation (n=9). *P<0.05 vs. JEZ-C, 0 µg/ml; ^#^P<0.05 vs. JEZ-C, 6.25×10^−3^ µg/ml..

**Figure 4.**
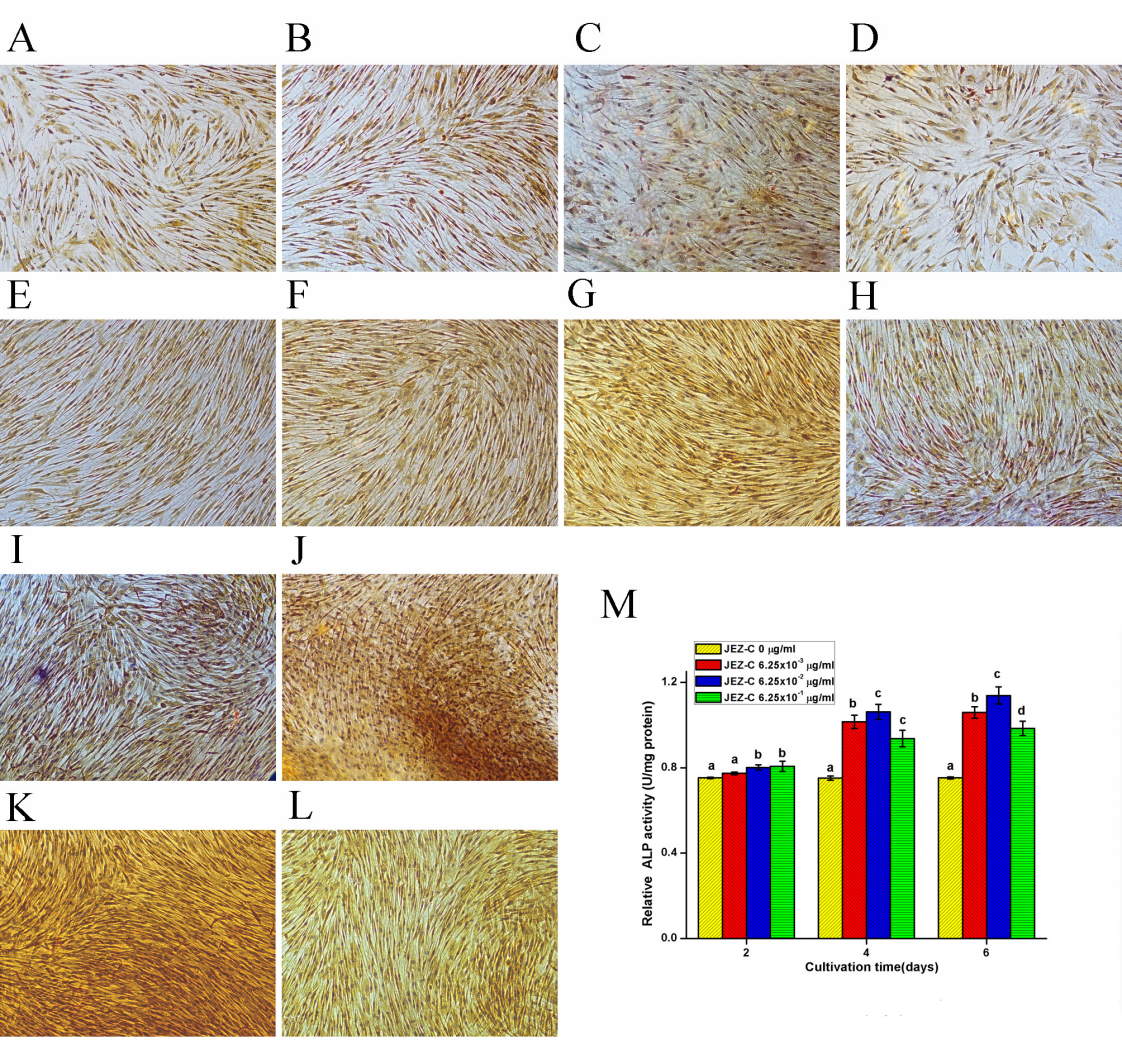
ALP staining of osteoblasts treated with various concentrations of JEZ-C. ALP staining of osteoblasts treated with (A) 0 µg/ml, (B) 6.25×10^−3^ µg/ml, (C) 6.25×10^−2^ µg/ml and (D) 6.25×10^−1^ µg/ml JEZ-C on day 2. ALP staining of osteoblasts treated with (E) 0 µg/ml, (F) 6.25×10^−3^ µg/ml, (G) 6.25×10^−2^ µg/ml and (H) 6.25×10^−1^ µg/ml JEZ-C on day 4. ALP staining of osteoblasts treated with (I) 0 µg/ml, (J) 6.25×10^−3^ µg/ml, (K) 6.25×10^−2^ µg/ml and (L) 6.25×10^−1^ µg/ml JEZ-C on day 6. Scale bar=100 µm. (M) Time-course of ALP activity of osteoblasts treated with various concentrations (0, 6.25×10^−3^, 6.25×10^−2^ and 6.25×10^−1^ µg/ml) of JEZ-C. Relative ALP activity (units/mg protein) was expressed as mean ± standard deviation (n=3). ALP activity in the 6.25×10^−2^ µg/ml JEZ-C group was significantly higher than in the other groups. *P<0.05 vs. JEZ-C, 0 µg/ml; ^#^P<0.05 vs. JEZ-C, 6.25×10−3 µg/ml. ALP, alkaline phosphatase.

**Figure 5.**
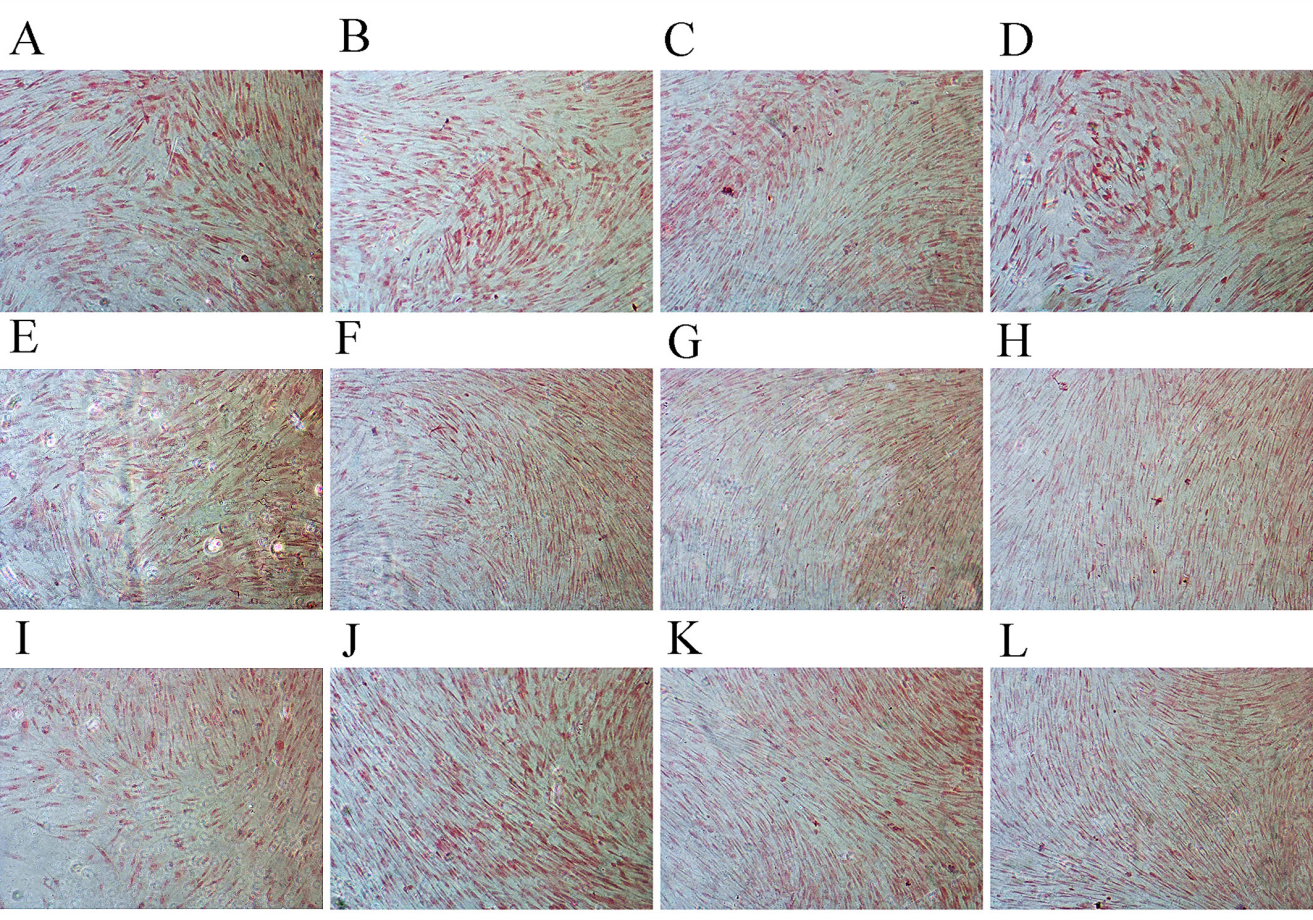
Alizarin red staining of osteoblasts cultured with various concentrations of JEZ-C over time. Staining of osteoblasts treated with (A) 0 µg/ml, (B) 6.25×10^−3^ µg/ml, (C) 6.25×10^−2^ µg/ml and (D) 6.25×10^−1^ µg/ml JEZ-C on day 2. Staining of osteoblasts treated with (E) 0 µg/ml, (F) 6.25×10^−3^ µg/ml, (G) 6.25×10^−2^ µg/ml and (H) 6.25×10^−1^ µg/ml JEZ-C on day 4. Staining of osteoblasts treated with (I) 0 µg/ml, (J) 6.25×10^−3^ µg/ml, (K) 6.25×10^−2^ µg/ml and (L) 6.25×10^−1^ µg/ml JEZ-C on day 6. Scale bar=100 µm. Staining was markedly strengthened over time in all groups. Cells treated with JEZ-C exhibited stronger staining compared with the control, particularly when treated with 6.25×10^−2^ µg/ml JEZ-C.

